# Goodness-of-Fit Tests and Model Diagnostics for Negative Binomial Regression of RNA Sequencing Data

**DOI:** 10.1371/journal.pone.0119254

**Published:** 2015-03-18

**Authors:** Gu Mi, Yanming Di, Daniel W. Schafer

**Affiliations:** 1 Department of Statistics, Oregon State University, Corvallis, Oregon, United States of America; 2 Molecular and Cellular Biology Program, Oregon State University, Corvallis, Oregon, United States of America; University of East Piedmont, ITALY

## Abstract

This work is about assessing model adequacy for negative binomial (NB) regression, particularly (1) assessing the adequacy of the NB assumption, and (2) assessing the appropriateness of models for NB dispersion parameters. Tools for the first are appropriate for NB regression generally; those for the second are primarily intended for RNA sequencing (RNA-Seq) data analysis. The typically small number of biological samples and large number of genes in RNA-Seq analysis motivate us to address the trade-offs between robustness and statistical power using NB regression models. One widely-used power-saving strategy, for example, is to assume some commonalities of NB dispersion parameters across genes via simple models relating them to mean expression rates, and many such models have been proposed. As RNA-Seq analysis is becoming ever more popular, it is appropriate to make more thorough investigations into power and robustness of the resulting methods, and into practical tools for model assessment. In this article, we propose simulation-based statistical tests and diagnostic graphics to address model adequacy. We provide simulated and real data examples to illustrate that our proposed methods are effective for detecting the misspecification of the NB mean-variance relationship as well as judging the adequacy of fit of several NB dispersion models.

## Introduction

The negative binomial (NB) model has been widely adopted for regression of count responses because of its convenient implementation and flexible accommodation of extra-Poisson variability. Let *Y* represent a univariate count response variable and *X* a *p*-dimensional vector of known explanatory variables. Then an NB log-linear regression model specifies that the probability distribution of *Y* is NB with mean *μ* and dispersion parameter *ϕ*, with log(*μ*) = *X*′ *β* where *β* is a *p*-dimensional vector of unknown regression coefficients.

The NB distribution can be derived as a Poisson-gamma mixture model. For the conventional parameterization (which we refer to as NB2), suppose *υ* is a gamma-distributed random variable with E (*υ*) = *μ* and Var (*υ*) = *ϕμ* ^2^, and that *Y*∣*υ* ∼ Poisson (*υ*), then the marginal distribution of *Y* is NB with mean *μ* and variance *μ* + *ϕμ* ^2^ (see, for example, [1]). The NB2 probability mass function (p.m.f.) has the form:

$$\begin{array}{c} f\left(y|\mu ,\phi \right)=\frac{\Gamma (y+\theta )}{\Gamma (\theta )\Gamma (y+1)}{\left(\frac{\theta }{\mu +\theta }\right)}^{\theta }{\left(\frac{\mu }{\mu +\theta }\right)}^{y}, \end{array}$$

where *θ* = 1/*ϕ*.

Other NB parameterizations follow from different parameterizations for the gamma mixing distribution. A general form, called NBP [2, 3], follows from the assumption that the gamma variance is *ϕμ* ^*α*^, and has the same form of p.m.f. *f*(*y*∣*μ*, *ϕ*), but with *θ* replaced by *ϕ* ^−1^ *μ* ^2−*α*^. In this parameterization, E(*Y*) = *μ* and Var(*Y*) = *μ* + *ϕμ* ^*α*^. We note that (1) for identically distributed count variables the NBP distribution is over-parameterized, but in a regression setting it offers additional flexibility in mean-variance modeling, which is useful in the RNA sequencing (RNA-Seq) analysis that follows; and (2) NBP includes the well-known NB1 (*α* = 1) and NB2 (*α* = 2) parameterizations as well as others. Greene [2] specified the symbol “P” for our *α*, which is why this parameterization is called “NBP”.

RNA-Seq analysis [4] may be performed on biological units from any of the traditional forms of life science study, such as randomized experiments with multiple treatments and covariates, or observational studies with multiple observed explanatory variables. The response variable for each unit is a vector of relative frequencies, each of which is a measure of a gene’s (or an isoform’s) expression level. Although much of the statistical attention to RNA-Seq analysis has so far been focused on the two-group problem—and, therefore, on identification of differentially expressed genes—there is a clear need for regression analysis for identifying differential expression after accounting for other variables, and for identifying patterns of expression and differential expression as a function of explanatory variables.

Future statistical techniques might be derived for the multivariate regression on all genes simultaneously, but the problem is currently tackled by the simpler univariate regression on each gene individually, with appropriate attention to false discovery rate. The response for a single gene is the number of RNA-Seq reads corresponding to that gene (*Y*) out of a total number of reads for the particular biological unit (*s*). Although there is evidence that the “technical variability” in *Y*—meaning the variability in the RNA-Seq technical procedure repeated on a single biological unit—can be described by a Poisson distribution [5], the observed variability from multiple biological units in the same observational or experimental group is greater than Poisson (see, for example, [6, 7]). The gamma mixture of Poissons, as described above, is a conceptually appealing alternative because the gamma mixing represents “biological variability”. Practically, the NB model is both flexible and convenient.

The primary statistical challenge involves simultaneous regression fitting for tens of thousands of genes from fairly small numbers of biological samples (e.g., less than twenty). An important power and efficiency issue in this case involves the modeling of the NB2 dispersion parameter *ϕ*. Five possibilities, for example, are (1) *ϕ* is constant for all genes; (2) *ϕ* is allowed to differ between genes but is constant within gene under all conditions; (3) *ϕ* is allowed to differ for all gene/condition combinations; (4) *ϕ* is taken to be a function of *μ*; and (5) *ϕ* is taken to have a trend as a function of *μ*, but with some additional between-gene variability. More flexible models are much more likely to fit the data, of course, but at the expense of tens of thousands of nuisance parameters. If a more specific model fits, based on vastly fewer nuisance parameters, it could offer substantial power and efficiency gains (for improved “true discovery” rates of differential expression, for example). Because of the very large number of hypothesis tests performed in a single RNA-Seq study and the very large number of RNA-Seq studies being performed world-wide, even a small improvement in power can have an important impact on the overall rate of scientific learning from the RNA-Seq technology.

Traditional tools for model diagnostics in generalized linear models (GLM), such as deviance and Pearson residuals and goodness-of-fit (GOF) tests, are suitable for binomial and Poisson regression if the means are large, i.e., the adequacy of the normal and *χ* ^2^ null distributions for residuals and GOF test statistics, respectively, are justified under central-limit-theorem-like asymptotics rather than large sample size asymptotics [8]. Such GOF tests are not appropriate for small means (which are typical for the majority of genes in RNA-Seq analysis), and the theory for the null sampling distribution of the residuals and GOF test statistics does not extend to NB regression.

In this article, we propose a goodness-of-fit test statistic for NB regression based on Pearson residuals, and the calculation of a *p*-value using Monte Carlo-estimated null sampling distributions. The same simulations are used to estimate expected ordered residuals for an empirical probability plot. For RNA-Seq diagnostics, the GOF *p*-values from all genes are examined in a uniform QQ plot and combined via the Fisher’s combined probability test (Fisher’s method [9]).

## Background

### Dispersion Modeling

Let *Y*_*ij*_ denote an RNA-Seq read count for the *i* ^*th*^ gene (*i* = 1, ⋯, *m*) of the *j* ^*th*^ experimental or observational unit (*j* = 1, ⋯, *n*), and **X**_*j*_ the associated *p*-dimensional explanatory variable. Suppose *Y*_*ij*_ ∼ NB(*μ*_*ij*_, *ϕ*_*ij*_) where *μ*_*ij*_ is the mean and *ϕ*_*ij*_ is the dispersion parameter in the NB2 parameterization. Suppose also that

$$\log\left(\mu_{ij}\right)=\log\left(s_{j}\right)+\log\left(R_{j}\right)+\log\left(\pi_{ij}\right),$$

with $\pi_{ij}=\exp(X_{j}^{\prime }\beta_{i})$, where *s*_*j*_ is the library size (the number of RNA-Seq reads in the biological sample from unit *j*), and *R*_*j*_ is an optional normalization factor estimated beforehand [6, 10, 11] and treated as known. In this formulation, *π*_*ij*_ is the mean relative frequency of occurrence of RNA-Seq reads associated with gene *i*, which is taken to be the expression level of gene *i* associated with observational or experimental unit *j*.

We label some of the ways to model the nuisance parameters *ϕ*_*ij*_ as follows:

1. Genewise: *ϕ*_*ij*_ = *ϕ*_*i*_ (constant within each gene *i* across all conditions *j*), with *m* parameters for NB dispersion.
2. Common: *ϕ*_*ij*_ = *ϕ* (constant for all gene/condition combinations), with one parameter for NB dispersion.
3. NBP: log(*ϕ*_*ij*_) = *α*_0_ + *α*_1_ log(*π*_*ij*_), equivalent to assuming NBP response distribution discussed in Di *et al.* [7], with two parameters for NB dispersion.

We also introduce here a new approach, in which the dispersion parameter trend is quadratic on the log scale:

4 NBQ: log(*ϕ*_*ij*_) = *α*_0_ + *α*_1_ log(*π*_*ij*_) + *α*_2_ [log(*π*_*ij*_)]^2^, with three parameters for NB dispersion.

An important related method estimates the *ϕ*_*ij*_’s via non-parametric regression:

5 Non-parameteric: *ϕ*_*ij*_ is estimated in a first step as a smooth function of $\log\left({\hat{\phi }}_{ij}\right)$ on $\log\left({\hat{\mu }}_{ij}\right)$, and then treated as known in the second step of regression coefficient inference.

In addition, there are variants that use an average of trend and individually-estimated dispersion parameters, based on empirical Bayes considerations [12]:

6 Tagwise-common: *ϕ*_*ij*_ is estimated as a weighted average of the common and genewise estimates, based on empirical Bayes calculations.
7 Tagwise-trend: *ϕ*_*ij*_ is estimated as a weighted average of the non-parametric and genewise estimates, based on empirical Bayes calculations.

Methods for inference from the genewise, common, non-parametric, tagwise-common, and tagwise-trend approaches are available in the edgeR Bioconductor package [13, 14]. The non-parametric method is also available in the DESeq and NBPSeq packages [6, 15]. The NBP and NBQ approaches are implemented in NBPSeq [15, 16].

The details of estimation for these methods are important but are not relevant to the proposed diagnostic tools and so are not discussed here. The adequacy of the models for RNA-Seq data is not yet well understood. We wish to use the model diagnostic tools proposed in this article to judge the degree of fit of the various models on real RNA-Seq data—particularly the fit of simple parametric models for the trend of log(*ϕ*) as a function of *π* and the degree of noise, if any, about this trend, so that realistic robustness and power studies can follow.

**Fig 1:**
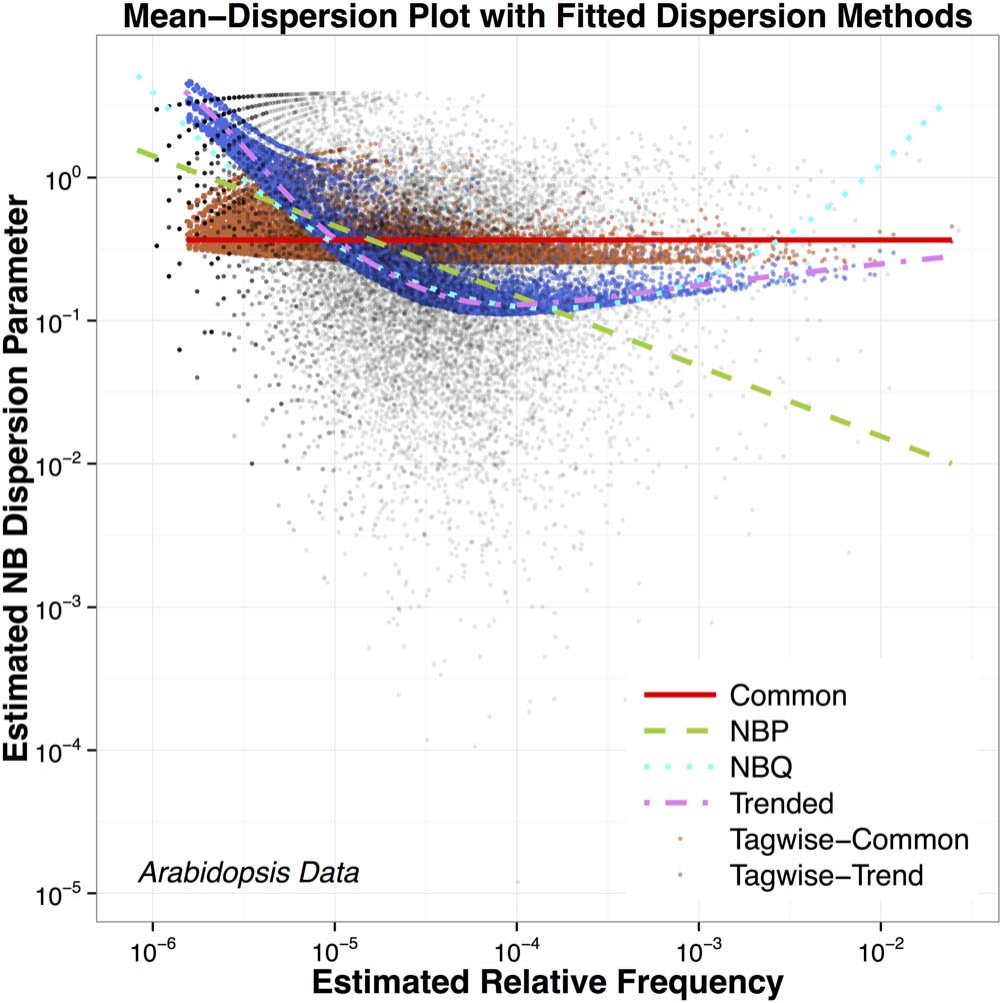
Mean-Dispersion Plot with Fitted Dispersion Models. The mean-dispersion plot with six fitted dispersion models (common, NBP, NBQ, trended, tagwise-common and tagwise-trend) for the Arabidopsis RNA-Seq dataset (19,623 genes from three biological samples in the mock treatment group). For better visualization, we use dots instead of curves for the tagwise procedures to indicate the variability of individual NB2 dispersion parameters about the trend.

To further clarify this point, Fig. 1 shows a log-log scatter plot of method-of-moments-like estimated NB2 dispersion parameters, $\hat{\phi }$, versus estimated mean relative frequencies, $\hat{\pi }$, for each of 19,623 genes from a single sample of size three of a pilot Arabidopsis RNA-Seq study examined in [7]. The curves on the plot are estimated dispersion trends based on the models described above. Polynomial gamma log-linear regression models of $\hat{\phi }$ on $\log(\hat{\pi })$ were used for quick-and-dirty testing and quantification of the trend, as follows. The linear model explains 24.1% of the variability in logged dispersion parameter estimates. A quadratic term (with *p*-value < 0.0001) explains an additional 7.2% of variability. A cubic term (*p*-value < 0.0001) in a full cubic model explains less than 0.1% additional variability. This plot and informal analysis suggest that the common *ϕ* model is inadequate; the trend is primarily, but not entirely, linear; and that a quadratic model captures essentially all of the trend in this particular dataset.

A simple model for trend in NB dispersion parameter *ϕ* as a function of mean relative frequency *π* is a good starting point for reducing the number of nuisance parameters, but the evidence of a trend does not imply that the *ϕ*’s fall exactly on the trend; there may be additional variability in *ϕ* for genes with the same value of *π*. The main questions we wish to address with diagnostic tools are the following: (1) Does the NB assumption hold for a very rich model (for both regression and dispersion)? (2) What relatively simple models are adequate for describing *ϕ* as a function of *π*? (3) Is there evidence of additional biological variability in *ϕ* between genes having the same value of *π*?

### Other Related Work on Model Diagnostics

Best *et al.* [17] extended Anscombe’s tests of fit for the NB distribution by using fourth order smooth tests, but these tests don’t extend in an obvious way to regression models for non-exponential family response distributions. The test we propose in this paper gives similar results to theirs for independent and identically distributed samples and can also be used for the procedures that involve non-parametric trend fitting and empirical Bayes averaging. Esnaola *et al.* [18] proposed a larger family of response distributions for RNA-Seq analysis, which permits the testing of NB as a special case; but we do not believe the approach (validated under extensively replicated experiments) is suitable for the small sample sizes we have in mind here.

Similar regression diagnostic approaches that use Monte Carlo or resampling to derive null sampling distributions of diagnostic quantities have been previously proposed for several situations. For ordinary linear regression, Atkinson [19] proposed half normal plots of jackknife residuals. For logistic regression, Landwehr *et al.* [20] proposed an “empirical probability plot” in which ordered residuals from the observed data are plotted against their expected values (or median values), as computed by Monte Carlo simulations. Their simulation procedure, which resembles parametric bootstrapping, is based on the estimated parameters from the fitted model. Similar graphical displays were adopted as informal checks of various count models. For example, Svetliza *et al.* [21] considered normal probability plots for log-linear Poisson, log-linear NB and non-linear NB models. Garay *et al.* [22] evaluated GOF between zero-inflated Poisson (ZIP) and zero-inflated NB (ZINB) models. Both of these used simulated envelopes in their plots, but with standard normal quantiles (instead of quantiles from simulations) on the *x*-axis. None of the aforementioned papers provided statistical tests for evaluating model lack-of-fit.

## Materials and Methods

### GOF Tests for Univariate NB Regression and the Empirical Probability Plot

We first consider univariate NB regression in this section and then return to the RNA-Seq problem of NB regression for each of many genes later. For regression data with counted response, we wish to determine whether any NBP model fits and, because of the convenience of NB2 estimation programs, whether the NB2 model fits in particular. We propose two GOF tests and an associated residual plot. The null hypothesis is that the count data follow the assumed NB regression model. In particular, the means follow the log-linear regression model, and the dispersion parameters follow the specified dispersion model (i.e., NB2, NBP, etc.). The test *p*-values provide an overall assessment of fit and the plot shows whether a small GOF *p*-value might be due to a small portion of the data. We use the same notation as in the “Background/Dispersion Modeling” subsection, but without the subscript *i*. In the RNA-Seq context, the methods of this section apply to a single gene. We start with Pearson residuals: $r_{j}\;=\;\left(y_{j}\;-\;{\hat{\mu }}_{j}\right)/{\hat{s}}_{j}$, where *μ̂*_*j*_ is the estimated NB mean and *ŝ*_*j*_ is the estimated NB standard deviation of *y*_*j*_ from the particular model being tested, for *j* = 1, ⋯, *n*.

**Fig 2:**
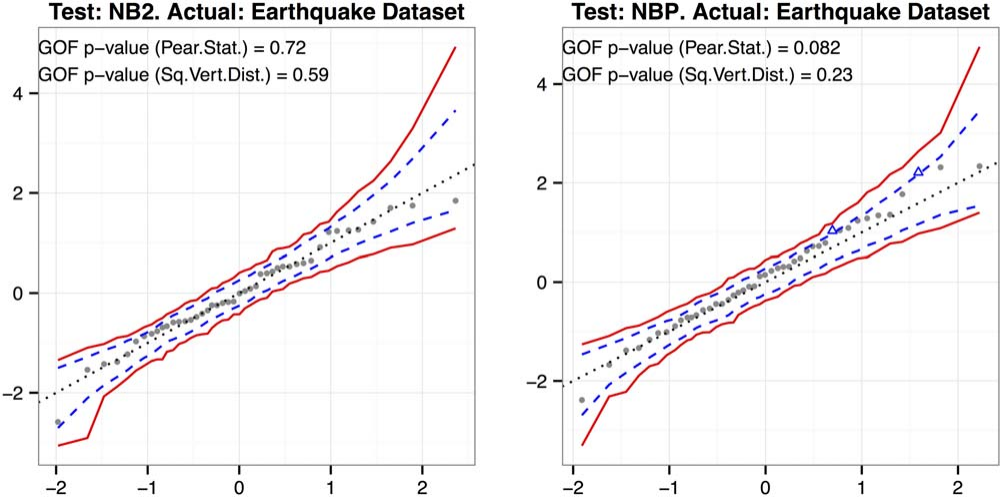
Empirical Probability Plots for Evaluating NB2 and NBP Model Fits. Empirical probability plots with GOF test *p*-values for evaluating NB2 and NBP model fits on the earthquake dataset (sample size: 45), with 95% prediction envelope in dashed blue lines and 95% simultaneous prediction band in solid red lines (based on 999 Monte Carlo simulations). Points outside the prediction envelope are flagged as blue triangles.

We first propose an empirical probability plot of the ordered Pearson residuals *r*_(*j*)_ versus the sampling distribution medians for each ordered Pearson residual, Med[*r*_(*j*)_], assuming the proposed NB regression model is correct. To approximate the medians, we simulate a large number of NB regression datasets of the same size and form as the observed one, using the data estimates as parameters for simulation; fit the same NB regression model to each simulated dataset; extract the ordered Pearson residuals; and retain the sample medians for each ordered residual. This is exactly the Landwehr *et al.*’s approach [20] applied to NB regression. A 95% pointwise prediction envelope (in dashed blue lines) can be formed from the similarly estimated 2.5^*th*^ and 97.5^*th*^ percentiles of the ordered residuals. Fig. 2 from the Results section shows an empirical probability plot for an earthquake event dataset.

We then wish to provide a global GOF test to accompany the empirical probability plot of Pearson residuals. A natural starting point is a test based on the Pearson statistic, i.e., the sum of squared Pearson residuals. The classical use of the *χ* ^2^ reference distribution is not appropriate here, but the null sampling distribution may be approximated by the Monte Carlo estimate. A *p*-value can be obtained as the proportion of simulated samples that produce a Pearson statistic as extreme or more extreme than the observed one. This is similar to the approach of Best *et al.* [17] in its application of parametric bootstrap to obtain a GOF *p*-value. We have found that our procedure and theirs give very similar results for samples with a common mean, but the approach discussed in Best *et al.* [17] requires estimation of higher order moments, which is difficult for the regression models we have in mind for RNA-Seq analysis.

Since the simulations provide estimated sampling distributions for each ordered residual, a finer test statistic is available as the sum of squared differences of the ordered residuals from their sampling distribution medians. We believed this was a worthwhile test statistic to consider given that we had already obtained approximate sampling distributions for each ordered residual to obtain the type of diagnostic plot discussed in Landwehr *et al.* [20]. The test is also related to the SAM graphical procedure of Tusher *et al.* [23] for identifying differential gene expression from microarray.

The following algorithm defines the diagnostic empirical probability plot of residuals and the Monte Carlo GOF test *p*-value based on the second test statistic.

#1 Fit an NB regression model from the data **Y** ^(0)^ = (*Y*_1_, ⋯, *Y*_*n*_)^*T*^; estimate all unknown dispersion parameters, e.g., ${\hat{\alpha }}_{0}$, ${\hat{\alpha }}_{1}$, ⋯, and regression coefficients ${\hat{\beta }}^{\left(0\right)}$; calculate Pearson residuals ${\text{r}}^{\left(0\right)}=(r_{1}^{\left(0\right)},\cdots ,r_{n}^{\left(0\right)})$ and the mean vector ${\hat{\mu }}^{\left(0\right)}$.
#2 For *h* = 1, ⋯, *R*:
  - Simulate a random vector **Y** ^(*h*)^ from $\text{NB}({\hat{\mu }}^{\left(0\right)},{\hat{\alpha }}_{0},{\hat{\alpha }}_{1},\cdots )$.
  - Compute and retain Pearson residuals **r** ^(*h*)^.
#3 Find the median, 2.5^*th*^ and 97.5^*th*^ percentiles of the Monte Carlo sampling distribution for each ordered residual, denoted by ${\tilde{r}}_{\left(j\right)}^{50}$, ${\tilde{r}}_{\left(j\right)}^{2.5}$ and ${\tilde{r}}_{\left(j\right)}^{97.5}$, respectively; plot the ordered residuals from the observed data against the Monte Carlo medians; draw a 95% pointwise prediction envelope on the plot by connecting the ${\tilde{r}}_{\left(j\right)}^{2.5}$’s (for lower bound) and the ${\tilde{r}}_{\left(j\right)}^{97.5}$’s (for upper bound).
#4 Compute the sum of squared deviations of ordered residuals from the medians of their sampling distributions $d^{\left(h\right)}=\overset{n}{\underset{j=1}{\sum }}\;{\left(r_{\left(j\right)}^{\left(h\right)}\;-\;{\tilde{r}}_{\left(j\right)}^{50}\right)}^{2}$ for the observed data (*h* = 0) and for the simulated samples (*h* = 1, ⋯, *R*); compute a Monte Carlo GOF test *p*-value by:
  $$\begin{array}{c} P_{1\text{sided}}^{MC}=\frac{{\sum^{}}_{h=1}^{R}\;𝟙\;\;\left(d^{\left(h\right)}\geq d^{\left(0\right)}\right)\;+\;1}{R\;+\;1}, \end{array}$$ (1)
  where 𝟙(*A*) is the indicator function equal to 1 if the event *A* is true and 0 otherwise [24].

The Pearson GOF *p*-value is computed in the same way, but using the sum of squared residuals as the test statistic. The two statistics are visualized on the empirical probability plots (see, e.g., Fig. 2) as the sum of squared deviations about the *y* = 0 line and the sum of squared deviations about the *y* = *x* dotted line. For this reason, we call the latter statistic the sum of squared vertical distances.

In using Monte Carlo simulation in lieu of theory to obtain the sampling distributions of the ordered residuals and test statistics, it would be ideal, but impossible, to use the *true* parameter values rather than their *estimates* from the data. Using estimated parameters in the simulation may lead to conservative test results, especially in small sample situations. As the sample sizes increase, we expect the sampling distribution of the *residuals* to be about the same.

We use *R* = 999 Monte Carlo samples so that the (binomial) standard error in *p*-value estimation is 0.016 for *p*-values near 0.5, 0.007 for *p*-values near 0.05, and 0.003 for *p*-values near 0.01. As pointed out in North *et al.* [25], adding 1 to both the numerator and the denominator in Equation (1) produces a slightly biased estimate of the true *p*-value but with the correct Type-I error rate, in contrast to the unbiased but anti-conservative *p*-value obtained without adding the 1.

### Diagnostic Tools for RNA-Seq Modeling

A major step for improving RNA-Seq analysis is the comparative evaluation of the models and methods for incorporating commonalities of NB dispersion parameters within and across genes, as described in the Introduction section and displayed in Fig. 1. For studying these models on a given RNA-Seq dataset, we propose fitting them, calculating the squared vertical distance NB GOF *p*-value for each of a randomly selected sample of genes (i.e., testing the univariate NB regression model fit for each gene individually, using the NB2 dispersion parameter estimated according to the particular dispersion model), drawing a uniform QQ plot of the *p*-values, and calculating a single *p*-value using the Fisher’s method.

Let *p*_*i*_ be the GOF *p*-value for gene *i*, based on the parameter estimates from the global model being tested. Fisher’s method produces a single GOF *p*-value by testing the conformity of the *p*_*i*_’s from *m* genes to a standard uniform distribution. If the *m* single-gene *p*-values are independent and all follow standard uniform distribution, the test statistic $X^{2}=-2\sum_{i=1}^{m}\log(p_{i})$ follows a $\chi_{\left(2m\right)}^{2}$ distribution. In this multiple genes case, the null hypothesis is that the NB counts for each gene follow the assumed NB regression model (the same as in the univariate case) and the NB dispersion parameters across all genes follow the specified dispersion model.

Although it is possible to base this on all genes, we elect to reduce the computational burden by selecting a random sample m* genes, and use m* = 1,000 as a computationally tolerable value. We do not have a direct way to study the suitability of this sample size for testing whether the *p*-values follow a uniform(0,1) distribution with Fisher’s method; but we do have an indirect approach that helps. Let *P* be the proportion of genes with *p*-values less than 0.05. A binomial 95% confidence interval for *P* from a sample of 1,000 genes has half-width 0.0135, so we would be likely to detect lack-of-fit to the uniform(0,1) if the actual proportion is 0.0635 or greater. Although we use Fisher’s method rather than this (arbitrary) binomial test, the binomial calculation provides some clarification of the type of departure from the uniform(0,1) that we are likely to detect with a sample of 1,000 genes. The *m* *p*-values are not exactly independent, as required for the theory of Fisher’s combined test, but are approximately so because the global parameter estimates are based on such a large number of genes.

As we noted earlier, the Pearson statistic can give misleading results if there are combinations of under- and over-dispersion relative to the response distributional model being tested. We have found this problem to be exacerbated in the RNA-Seq setting and so focus only on the squared vertical distance estimator, which does not suffer from the same problem.

An extremely small (or large) *X* ^2^ value can be due to either a small number of extreme single-gene *p*-values or a large number of moderately small (or large) *p*-values. The Fisher’s method itself cannot distinguish the different possibilities. Along with the Fisher’s combination of *p*-values, we suggest a uniform QQ plot of individual *p*-values to help reveal the nature of any lack-of-fit, indicated by a higher than expected proportion of small *p*-values. The proportion of genes with small *p*-values may have some effect on the thinking about appropriate models.

### The Earthquake Event Dataset

We consider an earthquake event dataset when illustrating the empirical probability plot and the GOF tests for univariate NB regression. The dataset (provided in the NBGOF package) contains the frequencies of all earthquakes of a given magnitude (reported to one decimal place) for magnitudes from 4.5 to 9.1, that occurred between January 1, 1964 to December 31, 2012 (Source: Composite Earthquake Catalog, Advanced National Seismic System, Northern California Earthquake Data Center (NCEDC), http://quake.geo.berkeley.edu/cnss/). The empirical probability plots with GOF test results, based on a log-linear regression of mean number of earthquakes on magnitude, are shown in Fig. 2. Neither the NB2 nor NBP model shows lack-of-fit.

### The Arabidopsis Study

We use an Arabidopsis dataset when demonstrating the diagnostic tools for RNA-Seq modeling. *Arabidopsis thaliana* has been intensively studied as a model organism in plant biology. The Arabidopsis data discussed in Di *et al.* [7] contain RNA-Seq reads that aligned to more than 25,000 genes from two groups of Arabidopsis samples of size three each. The two groups of size three each were derived from plants inoculated with Δ*hrcC* of *Pseudomonas syringae* pv tomato DC3000 or 10 mM MgCl_2_ (mock). The dataset used in this article comes from Di *et al.* [7], which is a subset of the data described in Cumbie *et al.* [26].

### Simulation Parameter Specifications

We specify the parameters in the simulation studies in the subsection “Results/Error Rates of GOF Tests in Simulations” (Tables 1 and 2) as follows: the mean is determined by *μ* = exp(*X*′ *β*) with the coefficient *β* = (15,−1.5). The design matrix *X* takes an intercept and a covariate equally spaced from 4 to 8 of length *n* = 5,10,50 and 100. The resulting mean levels approximately range from 20 to 8,100.

**Table 1:** Type-I Error Rate Evaluations.

			**Sq.Vert.D.**				**Pear.Stat.**			
**GOF Test For**	**Simulated Data**	**n:**	5	10	50	100	5	10	50	100
**NB2**	NB2		0.045	0.055	0.049	0.054	0.041	0.032	0.051	0.052
**NBP**	NB1		0.031	0.042	0.057	0.041	0.025	0.034	0.049	0.042
	NB2		0.040	0.034	0.056	0.060	0.044	0.030	0.047	0.044

Type-I error rates for 0.05-level NB2 and NBP GOF tests based on squared vertical distance (“Sq.Vert.D.”) or Pearson statistics (“Pear.Stat.”), from 1,000 simulated samples from each of several conditions. The standard error of simulation is approximately 0.007 for the Type-I error evaluations. The simulation conditions are detailed in the Materials and Methods section.

**Table 2:** Rejection Rate (Power) Evaluations.

			**Sq.Vert.D.**				**Pear.Stat.**			
**GOF Test For**	**Simulated Data**	**n:**	5	10	50	100	5	10	50	100
**NB2**	NB1		0.17	0.17	0.37	0.55	0.17	0.19	0.48	0.71
	NB2 + Outliers		0.05	0.13	0.45	0.70	0.03	0.15	0.56	0.84
	NB2 + Noise		0.06	0.12	0.33	0.54	0.05	0.08	0.13	0.17
**NBP**	NB2 + Outliers		0.07	0.18	0.74	0.95	0.08	0.15	0.84	0.99
	NB2 + Noise		0.05	0.06	0.36	0.61	0.05	0.04	0.16	0.22

Rejection rates for 0.05-level NB2 and NBP GOF tests based on squared vertical distance (“Sq.Vert.D.”) or Pearson statistics (“Pear.Stat.”), from 1,000 simulated samples from each of several conditions. The maximum standard error of simulation for the power evaluations is approximately 0.016. The simulation conditions are detailed in the Materials and Methods section.

For the NB2 model fit: the NB2 responses are simulated under *α*_0_ = log(0.1), *α*_1_ = 0 and *ϕ* = 0.1. The NB1 responses are obtained by simulating NB2 with *α*_0_ = log(0.5), *α*_1_ = −1, and *ϕ* = 0.5/*μ*. The “NB2 plus outliers” responses are simulated with the same dispersions as in NB2, except we randomly double 20% of responses (as outliers). The “NB2 plus noise” responses are simulated with the same dispersions as in NB2, except *ϕ* is specified as 0.1 ⋅ exp(*G*), where *G* ∼ 𝒩(0,1). The *α* in the variance *μ* + *ϕμ* ^*α*^ is determined by *α* = *α*_1_ + 2.

For the NBP model fit: the specifications are almost the same as in the NB2 model fit above, except for the simulated NB2 data, we use *α*_0_ = log(0.05) so that *ϕ* = 0.05.

## Results

### GOF Tests for Univariate NB Regression and the Empirical Probability Plot

#### Application to the Earthquake Dataset

This paper is mainly about RNA-Seq analysis, but in order to convey the univariate version of the problem, we wish to use a non-biological example, the earthquake dataset, that better illustrates the main issues (because of its larger sample size). Fig. 2 shows the empirical probability plot, along with a 95% pointwise prediction envelope (in dashed blue lines), for the NB2 and NBP regression of 48-year earthquake frequencies on magnitude (i.e., the Gutenberg-Richter Law, for 45 magnitudes from 4.5 to 9.1). Note that the band is formed from prediction intervals for the corresponding sample quantiles (not confidence intervals for their expected values). If the model fits, we would expect about 95% of the ordered residuals to fall within the band. We also superimpose a 95% simultaneous prediction band in solid red using the simulation method discussed in Buja *et al.* [27], so that for 95% of samples *all* ordered residuals should be contained in the red band. See the Materials and Methods section for details of the earthquake dataset.

The *p*-values for testing the NB2 and NBP models on the earthquake dataset are shown in Fig. 2. The suggestive Pearson statistic *p*-value for goodness-of-fit of the NBP model is due to two outliers, corresponding to the frequencies of earthquakes of magnitudes 7.1 and 7.8. Although NB2 (with variance function *μ* + *ϕμ* ^2^) and NBP (in which the variance function is estimated to be *μ* + *ϕμ* ^2.5^) produce nearly identical fits, the standard errors (in the denominators of the Pearson residuals) from the NBP fit tend to be smaller for the smaller counts, which is why the NBP, but not the NB2 diagnostic, is detecting some potential lack-of-fit of the simple log-linear model in the region of magnitudes between 7 and 8 (corresponding to relatively small frequencies).

#### Illustration on Simulated Datasets with Known Response Distributions

**Fig 3:**
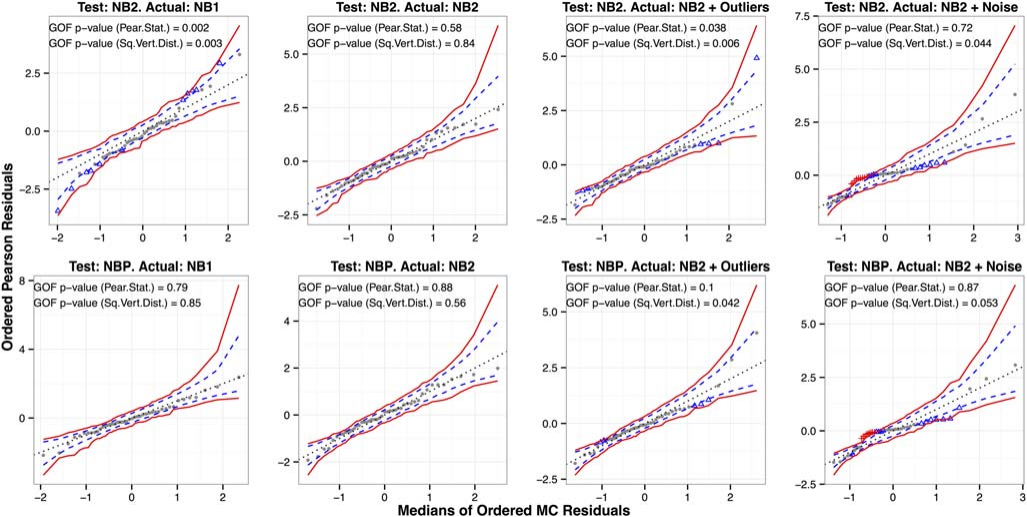
Empirical probability plots and GOF *p*-values for testing NB2 (top row) and NBP (bottom) on four simulated datasets with sample size = 45. The simulated response distributions are (left to right): NB1, NB2, NB2 with outliers and NB2 with random 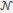
(0, 2^2^) noise added to log(*ϕ*). We superimpose 95% prediction envelopes in dashed blue lines and 95% simultaneous prediction bands in solid red lines (based on 999 Monte Carlo simulations). Points outside the prediction envelope but inside the simultaneous confidence bands are flagged as blue triangles, and points outside the simultaneous confidence bands are flagged as red crosses.

In Fig. 3 we demonstrate the NBP and NB2 empirical probability plots and Monte Carlo GOF test *p*-values on four simulated regression datasets with known response distributions. The regression structure is taken to be the estimated log-linear model from the earthquake dataset of Fig. 2, with sample size 45. For the first two scenarios, we generate NBP responses (with variance function *μ* + *ϕμ* ^*α*^) as follows: (1) NB1, with variance 2*μ* and (2) NB2, with variance *μ* + 0.1*μ* ^2^. For the third scenario, we simulate NB2 responses as (2) above, but introduce “outliers”: (3) “NB2 + Outliers”, by randomly doubling three of the 45 counts. For the last one, we generate a mixture of NB2 distributions with different dispersion parameters: (4) “NB2 + Noise”, with conditional variance *μ* + [0.1exp(*G*)] ⋅ *μ* ^2^, where *G* ∼ 𝒩(0, 2^2^). In this case the response counts are still gamma mixtures of Poissons, but the gamma variances are not constant.

In general, the two tests correctly indicate or fail to indicate lack-of-fit. An exception is that the Pearson test doesn’t do as well at detecting the lack-of-fit in the last two columns. As evident in the last column of the empirical probability plots in Fig. 3, the ordered Pearson residuals are larger in magnitude than expected in some regions and smaller in others. While the ordered residuals do not seem to behave as a sample from the tested distribution, the sum of their squares is moderated by the combination of small and large magnitudes. We are particularly concerned about this cancellation aspect of the Pearson test in the extension to RNA-Seq data, in which both under- and over-dispersion (relative to the tested model) may be present in subsets of genes. This issue is not relevant to the squared vertical distance test.

#### Error Rates of GOF Tests in Simulations

Table 1 shows the Monte Carlo Type-I error rates for 0.05-level tests using the NB2 and NBP Monte Carlo GOF tests on 1,000 simulated samples of NB1 and NB2 response distributions. The parameter specifications are detailed in the Materials and Methods section. The standard error of simulation is approximately 0.007. The Type-I error rates are smaller than the nominal values for both tests at the small sample sizes. As the sample size increases, the Monte Carlo evidence is consistent with actual Type-I error rates matching the nominal values. The severity of the small-sample conservatism is slightly greater for the Pearson test than for the squared vertical distance test.

Table 2 shows the estimated statistical power of the NB2 and NBP Monte Carlo GOF tests under several alternative distributions. In the “NB2 plus noise” alternative, we add random 𝒩(0,1) noise to log(*ϕ*) as described in the previous subsection, which means the data are a mixture of negative binomials with different dispersion parameters *ϕ*. In the “NB2 plus outliers” alternative, we randomly double 20% of the counts. The details of the generated distributions are also provided in the Materials and Methods section. The results do not indicate major power differences between the two tests, but the squared vertical distance test is more powerful in detecting the “NB2 plus noise” alternative.

### Diagnostic Tools for RNA-Seq Modeling

In this section, we apply our proposed GOF test and diagnostic graphics to the analysis of an Arabidopsis RNA-Seq dataset.

**Fig 4:**
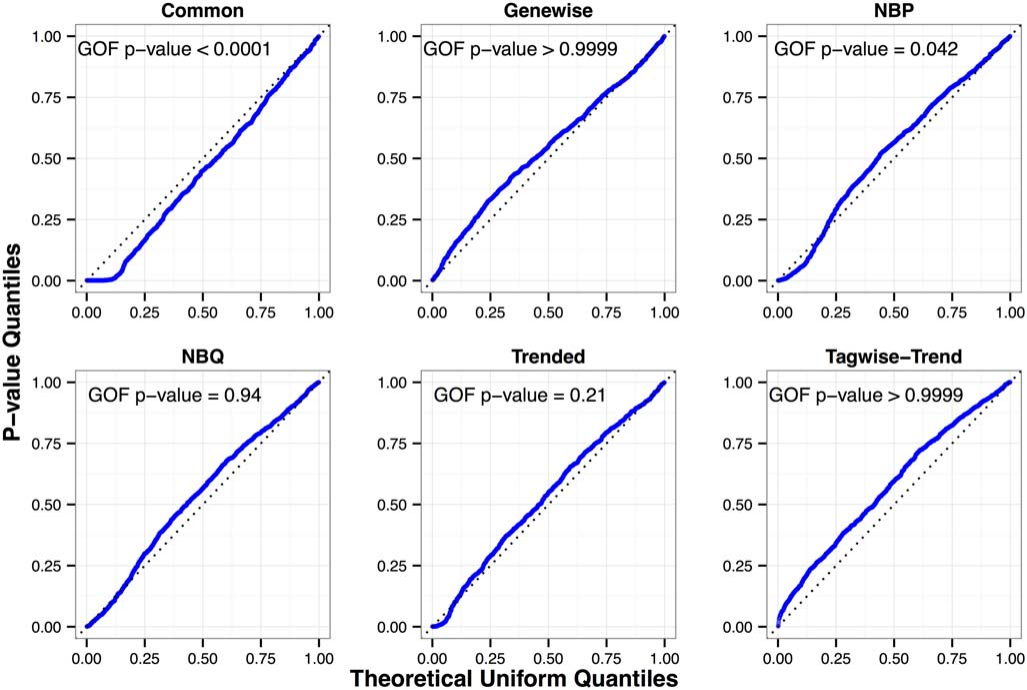
Uniform QQ plots of individual GOF test *p*-values for the Arabidopsis dataset. The results are based on a random sample of 1,000 genes from six experimental units in two experimental groups. The tagwise-common model (not shown) has a very similar pattern to the tagwise-trend model.

The following are the GOF *p*-values (in parentheses) from fitting the seven dispersion models (described in the “Background/Dispersion Modeling” subsection) to a random sample of 1,000 genes: common (< 0.0001); NBP (0.04); NBQ (0.94); trended (0.21); genewise (> 0.9999); tagwise-common (> 0.9999) and tagwise-trend (> 0.9999). The corresponding uniform QQ plots of *p*-values are shown in Fig. 4.

The *p*-values are greater than 0.9999 for the genewise and tagwise models. These unusually large *p*-values indicate that our GOF test may be conservative for these models. We note that for these models, the number of dispersion parameters to be estimated increases with the number of genes. In this sense, it is inherently more challenging to judge goodness-of-fit for these models. As can be seen in the uniform QQ plots of single-gene *p*-values, there are fewer small *p*-values than expected from a uniform(0,1) distribution. The extra large Fisher’s *p*-value is most likely due to conservativeness in the tests from small sample sizes, as evident in Table 1, when individual (genewise and tagwise) dispersion parameters are estimated from the small sample. Even a slight degree of conservativeness in the individual NB GOF tests can produce a very small Fisher combination statistic when there are so many *p*-values being combined. The evidence from Table 1, and our experience with simulations and other RNA-Seq datasets, suggest that this conservativeness diminishes with increasing sample size.

The consistency of the data with the NBQ trend model and the non-parametric “trended” approach suggests that models without noise about the trend may be adequate. We need to exercise caution with this conclusion, though—since the lack of evidence for lack-of-fit does not prove “fit” and that the test may be conservative at this sample size. Nevertheless, the apparent fit of NBQ in conjunction with the evidence of lack-of-fit for NBP is intriguing.

Fig. 1 shows the mean-dispersion plot (log-log scale) with six fitted dispersion models (common, NBP, NBQ, trended, tagwise-common and tagwise-trend) based on the mock treatment group alone. The genewise estimates are not included since there is no implied trend associated with that method.

In addition to the real RNA-Seq data analyses, we also performed simulation studies where the datasets are generated according to the “NB2 + noise” model. We then tested for GOF of the simple models and the tagwise approaches. Details on the simulation specifications and the results are provided in Supporting Information S1 File. The simulation results are as expected from the simulation setting.

## Conclusion and Discussion

In this article, we proposed a simulation-based GOF test and associated graphical displays for assessing NB model adequacy for NB regression, and we showed a way to combine those tests from multiple genes or gene isoforms in RNA-Seq datasets. We believe the results may be useful for ordinary regression with count responses, but our concentration is on the RNA-Seq setting.

We are interested in the potential power and efficiency gains in inferences from NB regression fits of individual genes when we adopt a global model that reduces the number of NB nuisance parameters. In this article we proposed methodology for judging such models. It is important to understand that there are two kinds of trend models relating the NB2 dispersion parameter to the mean. In one, represented by the NBP approach, the NB2 dispersion parameter is taken to be a simple function of the mean, so that the NB2 dispersion parameter will differ on the same gene for observations in different treatment groups if there are different expression levels in the different groups. For the trended approach and the related non-parametric approach in the DESeq and NBPSeq packages [6, 15], the NB2 dispersion parameter is taken to be constant for all observations on a single gene and that constant dispersion parameter is thought to be a smooth function of the average of means for that gene. It is not theoretically obvious whether the NB2 dispersion parameter should or should not be constant for a gene or, for that matter, whether the observed trend in dispersion parameter as a function of the mean is exact. We intend that the diagnostic analysis, performed on a variety of RNA-Seq datasets, will help provide an empirical clarification. The resolution of model adequacy is not, of course, the final piece of the puzzle. As in data analysis more generally, we do not expect models to fit exactly; we just need them to fit well enough for accurate and efficient inference. The diagnostic tools should help clarify models so that more comprehensive robustness and power studies can be used to compare the usefulness of the various inferential procedures upon which they are based.

The NBP model—in which the log of the NB2 dispersion parameter is a straight line function of the log of the mean—does not fully capture the trend in the RNA-Seq data we have examined. For that reason, we introduced the NBQ to allow for the next simplest model. We see evident improvement in model fit to the Arabidopsis data when the quadratic term is included. Note that the NBQ model also avoids the need for user-specified tuning parameters. Although the results for NBQ on the Arabidopsis data are intriguing, no strong generalizations about model adequacy emerge from the analysis of this single dataset or from the simulations based on the conditions of the dataset, which includes a very small sample size.

We are currently applying the diagnostic tools to a variety of RNA-Seq studies on different organisms. In that regard, we believe a useful picture emerges from the following set of diagnostic tools: (a) A plot of estimated NB2 dispersion parameter estimates with various model fits (as in Fig. 1). (b) The informal gamma log-linear regression analysis associated with that plot, including successive testing of polynomial terms and estimates of the proportion of variation in dispersion parameter estimates explained by polynomial models (as discussed in the “Background/Dispersion Modeling” subsection). (c) The NB GOF *p*-value from the fits to various models, such as the seven models reported in the real data example. (d) The estimate of *σ* in the noise model, in which the log of the NB2 dispersion parameter is the sum of a trend component (from NBQ trend or from trend estimated non-parametrically) and an individual component from a 𝒩(0, *σ* ^2^) distribution (as a measure of “noise” about the trend). In Mi and Di [28], we proposed a method for estimating *σ* ^2^ and studied the connection between magnitude of *σ* ^2^ and the performance of different DE test methods. Similar noise models had also been discussed in Wu *et al.* [29].

Finally, the goodness-of-fit of a dispersion model is only one of the factors that will affect the DE analysis. If a simpler parametric or nonparametric dispersion model shows good fit, it is a good indication that potential power can be saved in DE tests by using methods that “borrow information” between genes. When performing the DE tests, one still needs to carefully account for the uncertainty in the estimated dispersion model or dispersion parameters. The discussion of such DE test methods is beyond the scope of this paper. We refer readers to, for example, Lund *et al.* [30] and Love *et al.* [31], and corresponding software packages QuasiSeq and DESeq2.

### Software Information

The proposed approach is implemented as an R package named NBGOF (version 0.1.6, available in the Unix-like platforms) released at the first author’s github page: https://github.com/gu-mi/NBGOF, under GPL-2 License. The package also includes all datasets analyzed in this article. The R codes for reproducing all results in this article are available at the first author’s github page.

## Supporting Information

S1 File Supplementary Information for Simulation Results. Description of “NB2 + Noise” Simulated Datasets and S1 Fig. (uniform QQ plots of individual GOF test *p*-values for the simulated “NB2+noise” dataset) are provided in the Supporting Information S1 File.
(PDF)
