# Supplementary material for: Goodness-of-Fit Tests and Model Diagnostics for Negative Binomial Regression of RNA Sequencing Data
Source: PLoS One. 2015 Mar 18;10(3):e0119254. doi: 10.1371/journal.pone.0119254 (PMC4365073; doi:10.1371/journal.pone.0119254)
Supplement: S1 File — Description of “NB2 + Noise” Simulated Datasets and S1 Fig. (uniform QQ plots of individual GOF test p-values for the simulated “NB2+noise” dataset) are provided in the Supporting Information S1 File. (PDF) [file pone.0119254.s001.pdf]

Supporting Information for Goodness-of-Fit Tests and Model Diagnostics for Negative Binomial Regression of RNA Sequencing Data

Gu Mi[1,*], Yanming Di[1,2], Daniel W. Schafer[1]
1 Department of Statistics, Oregon State University, Corvallis, Oregon, United States of America
2 Molecular and Cellular Biology Program, Oregon State University, Corvallis, Oregon, United States of America
*** E-mail: neo.migu@gmail.com**

Description of "NB2 + Noise" Simulated Datasets and QQ Plots

In the "Results/Diagnostic Tools for RNA-Seq Modeling" section of the main text, we showed in Figure 4 the uniform QQ plots of GOF test p-values for different dispersion models fitted to the real Arabidopsis dataset. We also performed simulation studies in the multivariate setting to illustrate the performance of the tagwise methods as well as the simple models of common, NBP, NBQ, and trended. We simulated an RNA-Seq dataset according to the "NB2 + noise" model and applied the GOF test to the different dispersion models. Using the same 1,000 genes (as in the real data analysis) as a template, we specified μ according to the estimated β under the null model, and specified the NB dispersion parameter ϕ as

$$\phi = 0.1 \cdot \exp(\epsilon)$$

where $\epsilon \sim \mathrm{N}(0, 1)$. The read counts Y are then simulated from $\mathrm{NB}(\mu, \phi)$.

Figure S1 shows the uniform QQ plots of GOF test p-values for the common, NBP, NBQ, trended, tagwise-common and tagwise-trend models. The simple models of common, NBP, NBQ and trended all have p-values less than 0.05, while p-values greater than 0.9999 for tagwise approaches are likely due to the conservativeness explained in the manuscript (fewer small p-values than expected from a uniform(0,1) distribution from tests on the individual genes). The simulation results are as expected from the simulation setting.

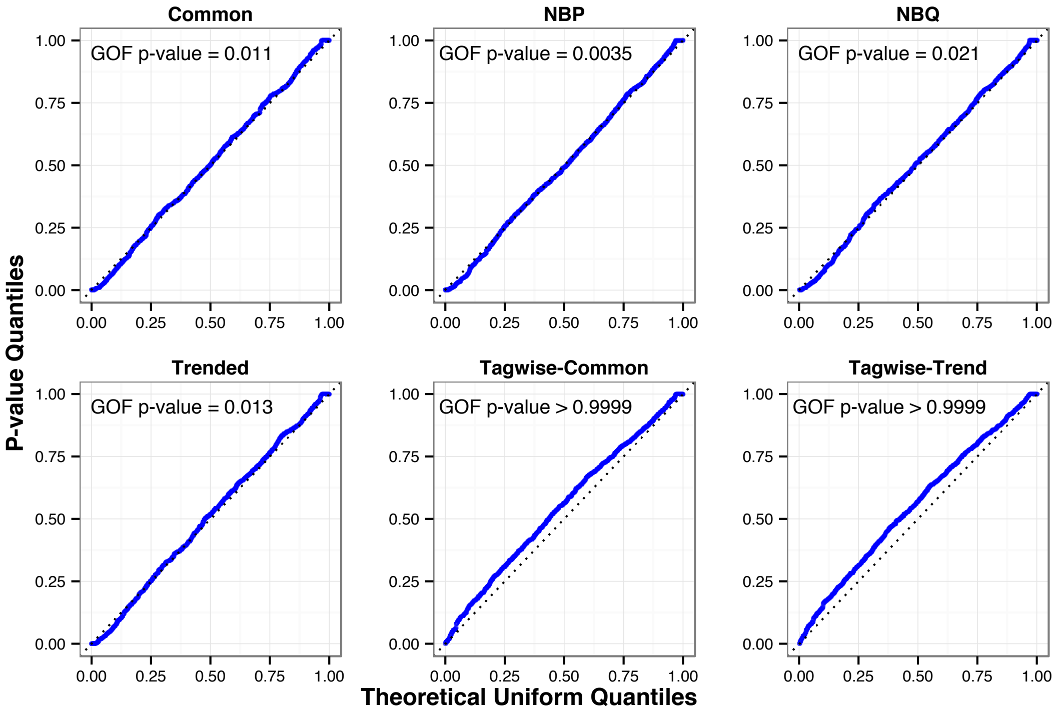

Figure S1. Uniform QQ plots of individual GOF test p-values for the simulated "NB2 + noise" dataset (use the Arabidopsis dataset as a template and base on a random sample of 1,000 genes from six experimental units in two experimental groups).
